# A pH Fingerprint Assay to Identify Inhibitors of Multiple Validated and Potential Antimalarial Drug Targets

**DOI:** 10.1021/acsinfecdis.3c00588

**Published:** 2024-03-18

**Authors:** Julia C. R. Lindblom, Xinxin Zhang, Adele M. Lehane

**Affiliations:** Research School of Biology, Australian National University, Canberra, Australian Capital Territory 2600, Australia

**Keywords:** malaria, Plasmodium falciparum, drug target, pH regulation, ion homeostasis

## Abstract

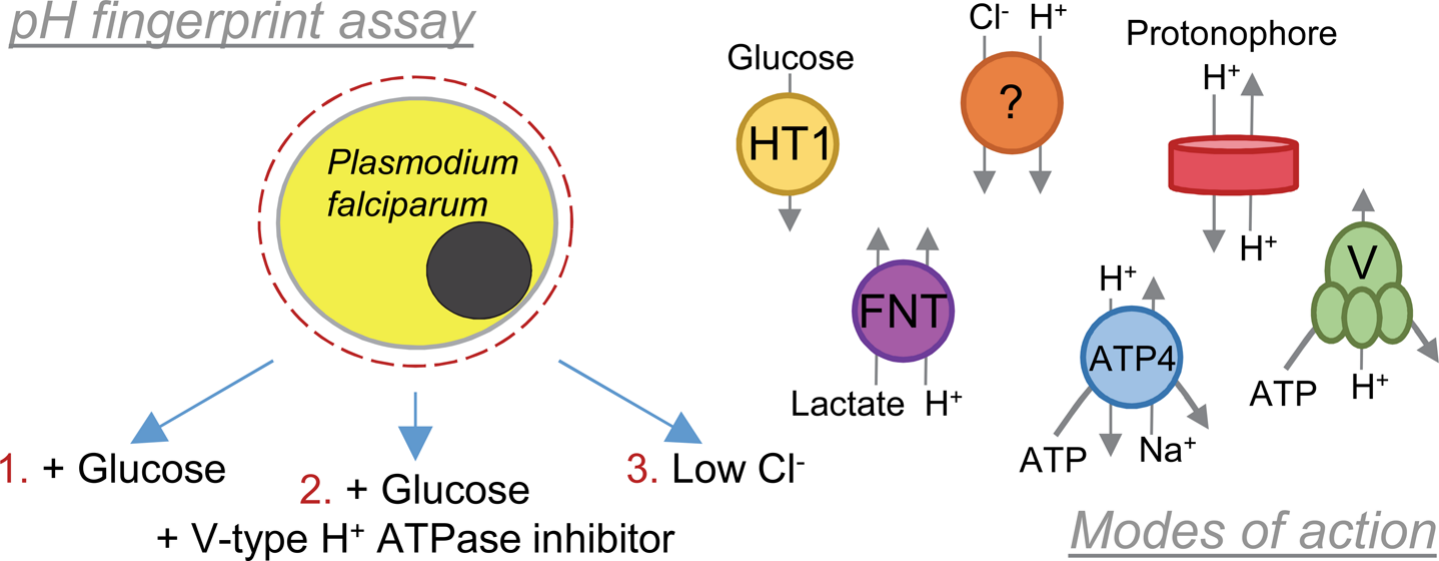

New drugs with novel modes of action are needed to safeguard malaria treatment. In recent years, millions of compounds have been tested for their ability to inhibit the growth of asexual blood-stage *Plasmodium falciparum* parasites, resulting in the identification of thousands of compounds with antiplasmodial activity. Determining the mechanisms of action of antiplasmodial compounds informs their further development, but remains challenging. A relatively high proportion of compounds identified as killing asexual blood-stage parasites show evidence of targeting the parasite’s plasma membrane Na^+^-extruding, H^+^-importing pump, PfATP4. Inhibitors of PfATP4 give rise to characteristic changes in the parasite’s internal [Na^+^] and pH. Here, we designed a “pH fingerprint” assay that robustly identifies PfATP4 inhibitors while simultaneously allowing the detection of (and discrimination between) inhibitors of the lactate:H^+^ transporter PfFNT, which is a validated antimalarial drug target, and the V-type H^+^ ATPase, which was suggested as a possible target of the clinical candidate ZY19489. In our pH fingerprint assays and subsequent secondary assays, ZY19489 did not show evidence for the inhibition of pH regulation by the V-type H^+^ ATPase, suggesting that it has a different mode of action in the parasite. The pH fingerprint assay also has the potential to identify protonophores, inhibitors of the acid-loading Cl^–^ transporter(s) (for which the molecular identity(ies) remain elusive), and compounds that act through inhibition of either the glucose transporter PfHT or glycolysis. The pH fingerprint assay therefore provides an efficient starting point to match a proportion of antiplasmodial compounds with their mechanisms of action.

## 1 Introduction

Progress in controlling malaria has stalled in recent years, with the estimated number of global cases remaining stubbornly high at 211–247 million per year between 2015 and 2021, and the number of deaths continuing to exceed 400,000 per year and recently surpassing 600,000.^1^ Drugs for preventing and treating malaria remain central to malaria control. Unfortunately, the parasite has shown a remarkable ability to acquire resistance to antimalarials, and this has compromised the utility of numerous drugs. Parasites resistant to the current first-line combination therapies are spreading.^2^ There is a shortage of treatment options in areas affected by multidrug resistance, and it is important that new drugs be developed so that faltering treatments can be replaced.

In recent years, millions of compounds have been screened for their ability to inhibit the proliferation of *P. falciparum* parasites (e.g., refs (3 and 4)). The process of determining mechanisms of action for the resulting “hits” has uncovered novel drug targets (e.g., refs (5–7)). Validating a new drug target typically involves a variety of approaches. A common starting point is “in vitro evolution” experiments, in which parasites are exposed to compounds of interest over a prolonged period of time. Often, resistant parasites emerge, and sequencing their genomes alongside those of their parents reveals the genetic basis of resistance. Mutation of the target is a common mechanism of resistance; thus, this approach often leads to a candidate target. In vitro evolution is a labor intensive approach. Even when successful, target confirmation requires the development of assays to study the activity of the target and its inhibition. Thus, determining the mechanisms of action of antiplasmodial compounds is a bottleneck in the drug discovery process, and thousands of promising antiplasmodial compounds are yet to be matched with their targets.

PfATP4, a protein on the parasite plasma membrane^8^ believed to serve as a Na^+^-extruding, H^+^-importing pump,^9^ has emerged as a highly vulnerable new drug target in recent years. PfATP4 belongs to a distinct subfamily of type II P-type ATPases, members of which are only found in apicomplexan parasites and their closest relatives.^10^ The “spiroindolones”^8^ were the first new class of antimalarial reported to act through inhibition of PfATP4^9^ and have been found to give rise to a range of readily detectable physiological perturbations including an increase in the parasite’s cytosolic Na^+^ concentration ([Na^+^]_cyt_),^9^ which is accompanied by osmotic swelling of the parasite and parasitized erythrocyte,^11^ and an increase in the parasite’s cytosolic pH (pH_cyt_).^9^ Since then, a large number of chemically diverse antiplasmodial compounds have been found to give rise to the physiological hallmarks of PfATP4 inhibition,^12−19^ including 7% of compounds in the Medicines for Malaria Venture’s (MMV’s) Malaria Box,^18^ and 9% of the compounds in the MMV’s Pathogen Box that were annotated as having antiplasmodial activity.^13^ The most clinically advanced PfATP4 inhibitor, cipargamin (a spiroindolone), has progressed well through multiple Phase 1 and 2 clinical trials.^20,21^

PfFNT is another *P. falciparum* plasma membrane transporter that has been validated as a drug target.^6,22^ It is the target of two structurally related compounds in MMV’s Malaria Box^6,22^—MMV007839 and MMV000972—as well as additional synthetic derivatives of them.^22,23^ The recent acquisition of high-resolution structures of PfFNT^24,25^ opens up opportunities for rational drug design. PfFNT belongs to the Formate Nitrite Transporter family, members of which are found in a broad range of microbes but not in animals.^26^ Under physiological conditions PfFNT exports H^+^ ions together with lactate.^26,27^ Like PfATP4 inhibitors, PfFNT inhibitors can be detected via their effects on a variety of physiological parameters including pH_cyt_.^6^ PfFNT inhibitors give rise to a cytosolic acidification.^6^ Thus, PfATP4 inhibitors and PfFNT inhibitors can both be detected and distinguished from one another via their (opposite) effects on pH_cyt_.

This finding prompted us to develop a pH-based assay that enables the simultaneous detection of inhibitors of as many transporters and processes as possible. In addition to inhibition of PfATP4 and PfFNT, there are a variety of other mechanisms by which parasite pH_cyt_ can be dysregulated, and indeed, a variety of antiplasmodial compounds that do not inhibit PfATP4 or PfFNT have also been shown to affect pH_cyt_.^6,13^

As in other cell types, the parasite’s resting pH_cyt_ is substantially higher than it would be if H^+^ were at electrochemical equilibrium across the plasma membrane and there is, accordingly, an inward H^+^ electrochemical gradient across the parasite plasma membrane.^28^ The multisubunit V-type H^+^ ATPase, located both on the parasite’s plasma membrane and on the membrane bounding its internal digestive vacuole (DV),^29^ plays a key role in maintaining a pH_cyt_ of ∼7.2–7.3^28,30^ as well as an acidic environment (pH ∼4.9–5.5^29−32^) in the parasite’s DV.^33^ Inhibition of the V-type H^+^ ATPase, for example with the well characterized specific inhibitors bafilomycin A1^34,35^ or concanamycin A,^36,37^ leads to an acidification of the parasite cytosol,^28^ an alkalinization of the DV,^33^ and parasite death.^38^ The V-type H^+^ ATPase is highly conserved among eukaryotes including humans, and it is not yet clear whether molecules that selectively target the parasite’s V-type H^+^ ATPase and are nontoxic to humans can be developed. Low-level resistance to an antimalarial candidate—the triaminopyrimidine ZY19489 (also known as MMV674253), which is undergoing testing in humans^39^—was associated with a mutation in the D subunit of the parasite’s V-type H^+^ ATPase, which raised the possibility that the V-type H^+^ ATPase is the target of this molecule.^40^ Furthermore, mutations were observed in the A subunit of the V-type H^+^ ATPase in parasites selected for resistance to the antiplasmodial compound SQ109.^41^ However, further studies are required to determine whether these molecules target the V-type H^+^ ATPase.

The V-type H^+^ ATPase hydrolyses ATP in transporting H^+^ “uphill” against its electrochemical gradient. Thus, compounds that deplete ATP will indirectly inhibit the V-type H^+^ ATPase (along with other ATPases including PfATP4) and cause a loss of the plasma membrane H^+^ gradient (a decrease in pH_cyt_). Blood-stage *P. falciparum* parasites generate ATP primarily via glycolysis.^42^ Thus, molecules that inhibit the glucose transporter on the parasite plasma membrane (the *P. falciparum* hexose transporter; PfHT^43^), or one of the parasite’s glycolytic enzymes, have the potential to deprive the parasite of ATP, with consequences for pH_cyt_.

PfHT has been of interest as a drug target for decades.^44^ The transporter has homology to human glucose transporters; however, the recent acquisition of high-resolution structures of PfHT^45,46^ opened up possibilities for rational drug design, leading to the design of potent and selective inhibitors of PfHT with submicromolar parasite killing activity.^47^

There is physiological evidence for an acid-loading Cl^–^ transporter on the parasite plasma membrane that plays a role in pH regulation (aiding parasite recovery from an alkalinization event) and allows the parasite to maintain a cytosolic Cl^–^ concentration higher than what would be expected if Cl^–^ were at electrochemical equilibrium.^48^ The molecular identity of the acid-loading Cl^–^ transporter is not known, and it is possible that more than one transporter is involved.

A number of compounds in the MMV’s Malaria Box have been found to dissipate the H^+^ gradients across both the parasite’s plasma membrane and digestive vacuole membrane (acidifying the parasite cytosol and alkalinizing the parasite’s digestive vacuole).^6^ Compounds that exert these effects more rapidly than a supramaximal concentration of a V-type H^+^ ATPase inhibitor are likely to have protonophore activity (i.e., render membranes more permeable to H^+^).

**Figure 1:**
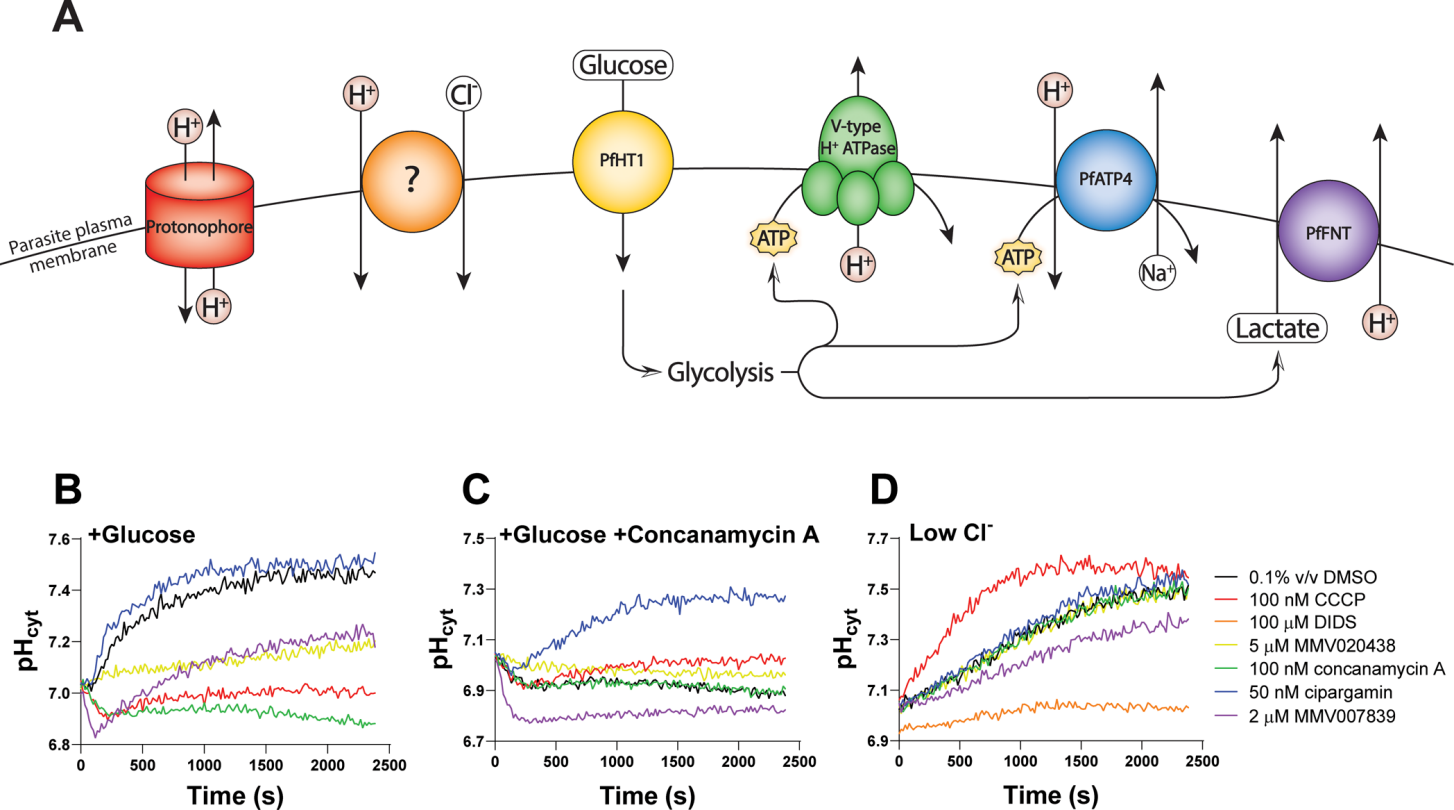
The pH fingerprint assay. (A) The pH fingerprint assay has the potential to detect (i) protonophores and inhibitors of (ii) the acid-loading Cl^–^ transporter(s) of unknown molecular identity, (iii) the glucose transporter PfHT/glycolysis, (iv) the V-type H^+^ ATPase, (v) PfATP4, and (vi) PfFNT. (B–D) pH traces in the +Glucose (B), +Glucose + concanamycin A (C) and Low Cl^–^ (D) conditions for 0.1% v/v DMSO (solvent control; black), the protonophore CCCP (100 nM; red), the general anion transport inhibitor DIDS [100 μM; orange; included only in (D)], the PfHT inhibitor MMV020438 (5 μM; yellow), the V-type H^+^ ATPase inhibitor concanamycin A (100 nM; green), the PfATP4 inhibitor cipargamin (50 nM; blue), and the PfFNT inhibitor MMV007839 (2 μM; purple). The data in panels (B–D) are from a single experiment with 3D7 parasites, representative of numerous experiments in which these control compounds have been included (see Figures 2–4 for additional experiments with 3D7, and Figure 5 for an experiment with different parasite strains).

Here, we sought to develop a pH-based assay that can serve as an efficient starting point to identify protonophores and inhibitors of PfATP4, PfFNT, the V-type H^+^ ATPase, PfHT/glycolysis, and the acid-loading Cl^–^ transporter(s) of unknown identity (Figure 1A). The assay entails measuring the pH-response of isolated ATP-depleted malaria parasites to compounds of interest, measured under three different experimental conditions. These three conditions differ from one another with regard to the presence or absence of the ATP-restoring nutrient glucose, the V-type H^+^ ATPase inhibitor concanamycin A, and the extracellular Cl^–^ concentration. In the process of designing and validating this assay, we gained new insights into ion regulation in the parasite and new information on the mode of action of the clinical candidate ZY19489.

## 2 Results

### 2.1 An Assay for the Simultaneous Detection of Protonophores and Inhibitors of Multiple Plasma Membrane Transporters

The input for the pH fingerprint assay is *P. falciparum* trophozoite-stage parasites that have been functionally isolated from their host erythrocytes, loaded with the ratiometric pH-sensitive dye BCECF, and depleted of ATP. pH_cyt_ is then measured in parasites suspended in three separate solutions: (1) A saline solution containing glucose (+Glucose), (2) A saline solution containing glucose and the V-type H^+^ ATPase inhibitor concanamycin A (+Glucose + concanamycin A), and (3) A glucose-free saline solution in which Cl^–^ has been replaced with gluconate (low Cl^–^).

#### 2.1.1 Control Compounds

The following control compounds are used in all experiments: the PfATP4 inhibitor cipargamin,^8,9^ the PfFNT inhibitor MMV007839,^6,22^ the V-type H^+^ ATPase inhibitor concanamycin A (shown previously to be active against the *P. falciparum* V-type H^+^ ATPase^38^), the PfHT inhibitor MMV009085^49,50^ or MMV020438 (also known as TCMDC-125163),^50^ and the well-characterized protonophore CCCP (shown previously to be effective in asexual blood-stage *P. falciparum* parasites^51^). The effects of each of the control compounds under the three different conditions of the pH fingerprint assay are described below. While there are no known specific inhibitors of the parasite’s acid-loading Cl^–^ transporter(s), the transporter(s) can be inhibited with the general anion transport inhibitor DIDS.^48^ In light of the lack of specificity of DIDS, it is included as a control only in the low Cl^–^ condition in which inhibitors of the acid-loading Cl^–^ transporter(s) would be expected to be identifiable.

#### 2.1.2 +Glucose Condition

When ATP-depleted parasites were placed in a saline solution containing glucose, they imported glucose via PfHT^43^ and generated ATP through glycolysis,^42^ allowing the activity of ATPases including the V-type H^+^ ATPase to resume.^28^ The activity of the V-type H^+^ ATPase allowed parasites to re-establish an inward H^+^ electrochemical gradient across the plasma membrane, and their pH_cyt_ increased over time until a resting pH_cyt_ of ∼7.4 was obtained [Figure 1B; solvent control (0.1% v/v DMSO) trace (black)]. The V-type H^+^ ATPase inhibitor concanamycin A prevented the restoration of the H^+^ gradient across the parasite plasma membrane (Figure 1B, green trace). In fact, the cytosol acidified slightly, likely as a result of H^+^ loading by PfATP4,^52^ which is expected to have become active again once glucose was restored and ATP production resumed. The PfHT inhibitor MMV020438, when tested at 5 μM, slowed down the rate of alkalinization, consistent with it partially inhibiting (and thereby slowing) glucose import (Figure 1B, yellow trace). The protonophore CCCP prevented an inward H^+^ gradient from being re-established, consistent with it increasing the permeability of the plasma membrane to H^+^ to an extent that could not be effectively countered by the V-type H^+^ ATPase (Figure 1B, red trace). The PfFNT inhibitor MMV007839 initially gave rise to a decrease in pH_cyt_, as the restoration of glycolysis led to the production of lactate and H^+^ that would normally be effluxed via PfFNT. The pH then increased again (Figure 1B, purple trace). This was most likely a result of H^+^ efflux via the V-type H^+^ ATPase, as in the +Glucose + concanamycin A condition, little pH recovery post-acidification was observed (Figure 1C, purple trace). This biphasic effect of PfFNT inhibitors on pH_cyt_ in a glucose-containing solution after a period of ATP depletion has been reported previously.^6^ The PfATP4 inhibitor cipargamin gave rise to a slight increase in the rate of alkalinization (Figure 1B, blue trace), as expected from inhibition of the PfATP4-mediated movement of H^+^ in the opposite direction to that mediated by the V-type H^+^ ATPase. However, the effects of PfATP4 inhibitors were more pronounced in the +Glucose + concanamycin A condition.

#### 2.1.3 +Glucose + Concanamycin A Condition

Inhibitors of PfATP4 (Figure 1C, blue trace) and PfFNT (Figure 1C, purple trace) were readily identified in the +Glucose + concanamycin A condition. Despite the restoration of glucose, the solvent control parasites did not recover their pH_cyt_ to a normal resting value, as the V-type H^+^ ATPase was inhibited by concanamycin A (Figure 1C, black trace). The PfFNT inhibitor MMV007839 gave rise to an acidification (Figure 1C, purple trace), consistent with the lactate and H^+^ produced from glycolysis being unable to exit the parasite effectively. An increase in pH_cyt_ was observed in the presence of the PfATP4 inhibitor cipargamin (Figure 1C, blue trace), consistent with previous findings.^12^ PfATP4 is an “acid-loader”,^9,52^ and the increase in pH_cyt_ suggests that in this condition, when glucose was restored, the V-type H^+^ ATPase inhibited, and PfATP4-mediated H^+^ entry eliminated, more H^+^ ions were leaving the parasite or being consumed in the parasite than were entering or being produced. PfATP4 inhibitors have been shown previously to give rise to a cytosolic alkalinization in standard pH experiments in which parasites are continuously maintained in glucose-containing media,^9^ although this can be difficult to detect with a plate reader.^13^ In the +Glucose + concanamycin A condition of the pH fingerprint assay, PfATP4 inhibitors were reliably detected. In a blind screen of the antiplasmodial compounds within the Pathogen Box, all 11 previously reported PfATP4 inhibitors were identified based on their effects on pH_cyt_ in this condition (Figure S1).

As expected, the trace for the V-type H^+^ ATPase inhibitor concanamycin A (Figure 1C, green) overlapped with that for 0.1% v/v DMSO (Figure 1C, black), as concanamycin A was already present at a supramaximal concentration (100 nM) in the solution to which all of the parasites were exposed in this condition, so a further increase in concentration was without effect. In both cases, pH_cyt_ decreased somewhat from its starting value, likely because of PfATP4-mediated H^+^ loading. The traces for CCCP (protonophore; Figure 1C, red) and MMV020438 (PfHT inhibitor; Figure 1C, yellow) were slightly separated from those of 0.1% (v/v) DMSO and concanamycin A, although this was subtle and is not always detectable, and the effects of both of these control compounds were more pronounced in other conditions within the assay. In the case of MMV020438, PfATP4 activity (and therefore H^+^ loading) may have been reduced as a consequence of partial inhibition of glucose entry and, therefore, ATP production. In the case of CCCP, the data are consistent with an increased permeability of the plasma membrane to H^+^ preventing a H^+^ gradient (under these conditions, outward) being generated by PfATP4-mediated H^+^ loading.

#### 2.1.4 Low Cl^–^ Condition

Parasites underwent an alkalinization when suspended (at time 0) in a glucose-free solution with a low Cl^–^ concentration (the final extracellular Cl^–^ concentration was 14.2 mM). Under physiological conditions, the Cl^–^ transporter is believed to import Cl^–^ and H^+^ equivalents into the parasite.^48^ However, the transporter is bidirectional, and upon suspending parasites in a low Cl^–^ solution, an outward Cl^–^ gradient is created, leading to the export of Cl^–^ and H^+^ equivalents.^48^ The nonspecific anion transport inhibitor DIDS prevented the alkalinization from occurring (Figure 1D, orange trace), as reported previously,^48^ and consistent with the Cl^–^ transporter(s) being inhibited. This compound has also been shown to inhibit (radioactive) ^36^Cl^–^ transport across the parasite plasma membrane.^48^ The PfFNT inhibitor MMV007839 also decreased the rate of alkalinization in these conditions (Figure 1D, purple trace); this was unexpected and therefore explored further below. The protonophore CCCP increased the rate at which the cytosol alkalinized (Figure 1D, red trace). The basis for this requires further investigation (see Discussion).

In summary, when their effects on parasite pH_cyt_ were recorded under the three different conditions of the assay, the control compounds having different mechanisms of action (Figure 1A) each gave rise to unique “pH fingerprints” (Figure 1B–D). Having established this, we went on to test additional compounds known or suspected to have one of the modes of action shown in Figure 1A to validate the assay. We also tested the clinical candidate ZY19489 to investigate whether it targets the V-type H^+^ ATPase.

### 2.2 Validating the pH Fingerprint Assay through the Testing of Additional Compounds

Compounds that do not have any of the modes of action that can be detected with the pH fingerprint assay are expected to give rise to traces that overlap with those of the solvent control (0.1% v/v DMSO). Compounds with the same mode of action as cipargamin (PfATP4 inhibitor), MMV007839 (PfFNT inhibitor), concanamycin A (V-type H^+^ ATPase inhibitor), or CCCP (protonophore) are expected to produce traces that overlap with those seen in response to the addition of each of these agents if they are used at a concentration that has a maximal effect. If the test compounds are used at a submaximal concentration, their traces are expected to fall between those of the control compound with the same mode of action and those of the solvent control (0.1% v/v, DMSO) under conditions in which these are different. Specific examples are provided below.

**Figure 2:**
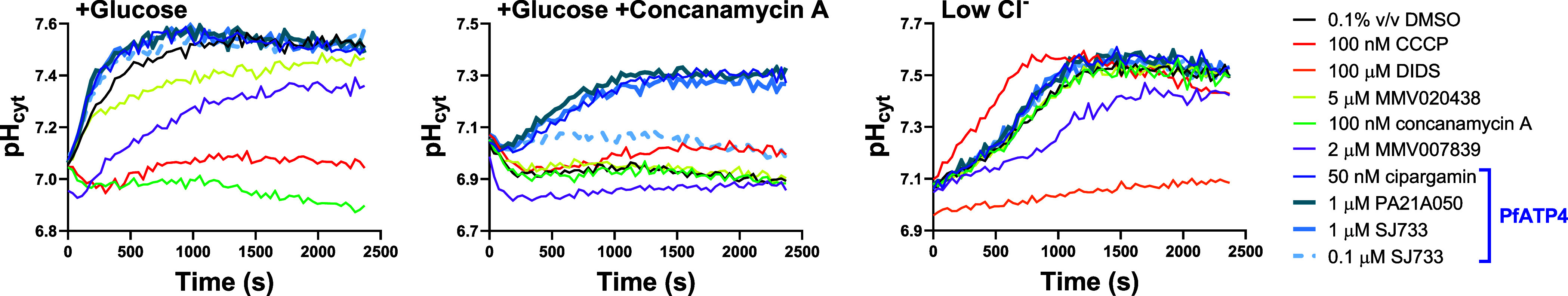
pH fingerprint of PfATP4 inhibitors when tested at maximal and submaximal concentrations. In addition to the control compounds described in Figure 1, the PfATP4 inhibitor SJ733 was tested in the three conditions of the pH fingerprint screen at 1 μM (a concentration that had a maximal effect; thick light blue traces) and 0.1 μM (a concentration that gave rise to submaximal inhibition of PfATP4; thick dashed light blue traces). The PfATP4-targeting compound PA21A050 was tested at 1 μM, a concentration that gave rise to a maximal effect (thick turquoise traces). The data are from a single experiment, representative of three similar experiments in which SJ733 and PA21A050 were tested (see Figure S2 for an additional experiment).

When tested at 1 μM, the known PfATP4-targeting antiplasmodial compounds SJ733^17^ and PA21A050^19^ gave rise to traces that overlapped with those of cipargamin (Figure 2, thick lines). When tested at a lower concentration (0.1 μM, ∼3-fold higher than its 50% inhibitory concentration (IC_50_) for inhibition of the growth of 3D7 parasites in 72 h assays^17^), SJ733 produced traces that fell between those of cipargamin and 0.1% v/v DMSO in the +Glucose and +Glucose + concanamycin A conditions (Figure 2, dashed lines). In the low Cl^–^ condition, traces for PfATP4 inhibitors (at maximal and submaximal concentrations) did not differ significantly from those of 0.1% v/v DMSO (Figure 2).

**Figure 3:**
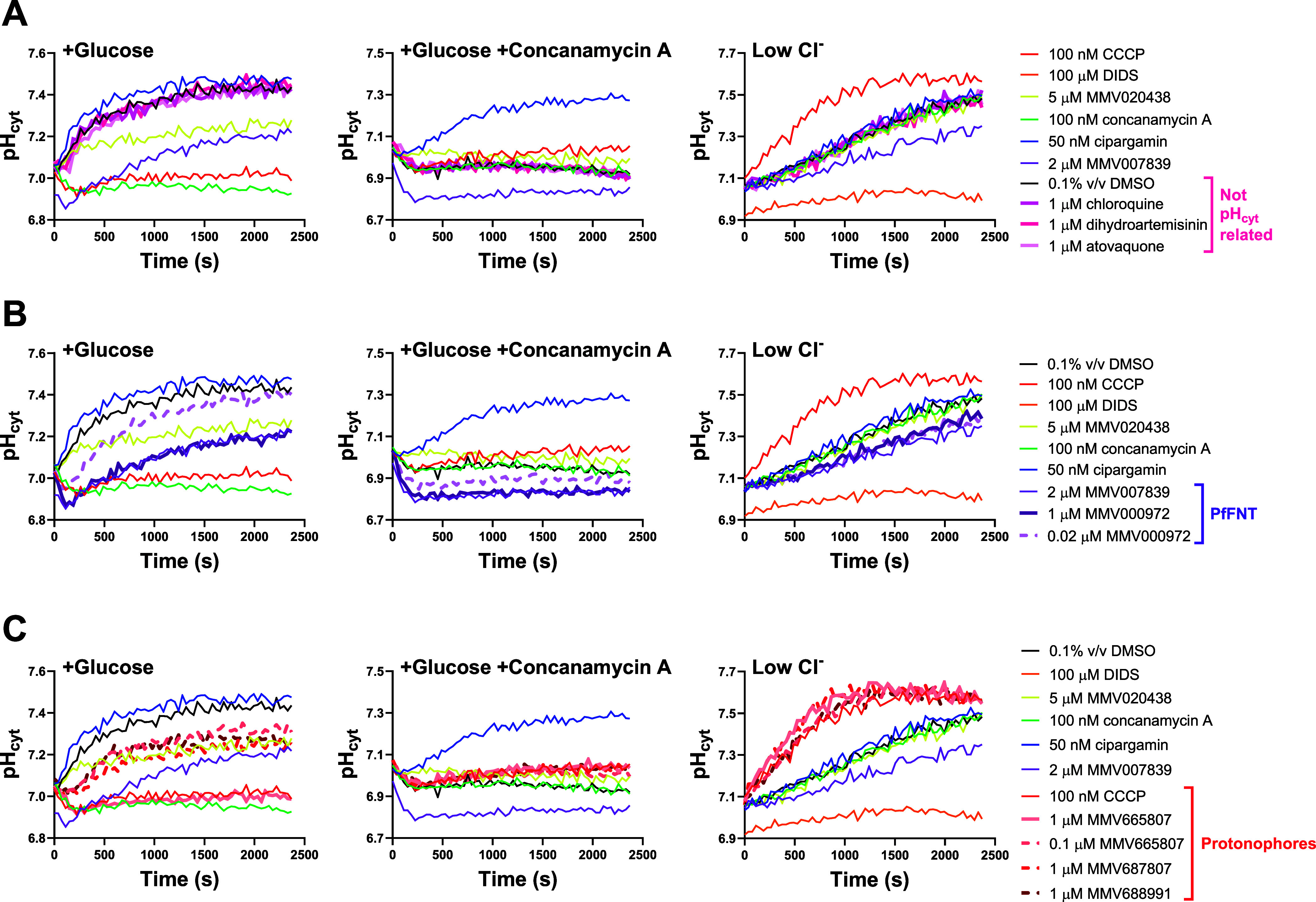
pH fingerprints of compounds that do not have any of the modes of action that can be detected with the assay (A), PfFNT inhibitors at maximal and submaximal concentrations (B), and suspected protonophores at maximal and submaximal concentrations (C). In addition to the control compounds described in Figure 1, results for the antimalarials chloroquine (thick pink traces), dihydroartemisinin (thick dark pink traces), and atovaquone (thick light pink traces), all tested at 1 μM, are shown in panel (A). Panel (B) shows results for the PfFNT inhibitor MMV000972, which was tested at concentrations that gave rise to maximal (1 μM; thick purple traces) and submaximal (0.02 μM; thick dashed purple traces) effects. Panel (C) presents the results for three suspected protonophores: MMV665807 [tested at 1 μM (thick peach traces), which had a maximal effect, and 0.1 μM (thick dashed peach traces), which had a submaximal effect], MMV687807 (1 μM; thick dashed red traces), and MMV688991 (1 μM; thick dashed dark red traces). The data shown in the three panels are from the same experiment; thus, the traces for the control compounds are identical in each panel. All of the test compounds have been tested in at least three independent experiments, yielding consistent results (see Figure S2 for an additional experiment for each compound).

The traces for three antimalarial compounds that do not have any of the mechanisms of action that can be detected with the pH fingerprint assay are shown in Figure 3A: chloroquine (thought to act primarily via inhibition of haem detoxification in the parasite’s digestive vacuole;^53−55^ thick pink lines), dihydroartemisinin (believed to damage multiple cellular components upon activation by haem-derived iron;^56,57^ thick dark pink lines), and atovaquone (an inhibitor of the cytochrome *bc*1 complex;^58^ thick light pink lines). The traces for each of the three compounds (each tested at 1 μM) overlapped with those of 0.1% v/v DMSO.

The effects of maximal (1 μM) and submaximal (0.02 μM) concentrations of a PfFNT inhibitor from the Malaria Box, MMV000972,^6,22^ are shown in Figure 3B. MMV000972 has an IC_50_ value for inhibition of parasite growth of 1.8 μM in 72 h assays (Dd2 strain^6^), yet has been shown to inhibit PfFNT in various in vitro assays at submicromolar concentrations.^6^ The traces for 1 μM MMV000972 (thick dark purple lines) overlapped with those of MMV007839 (a different PfFNT inhibitor from the Malaria Box used as the control; Figure 1) in all three conditions. The traces for 0.02 μM MMV000972 (dashed purple lines) fell between those of the MMV007839 and 0.1% (v/v) DMSO controls in the +Glucose and +Glucose + concanamycin A conditions. In the low Cl^–^ condition the trace for 0.02 μM MMV000972 was not readily distinguished from that of the MMV007839 control.

The traces of the three compounds that were suspected of having protonophore activity are shown in Figure 3C. MMV665807, a compound from the Malaria Box, was shown previously to acidify the parasite cytosol and alkalinize the DV.^6^ When tested at 1 μM (close to its IC_50_ value for inhibition of the growth of 3D7 parasites^59^), the traces for MMV665807 (thick peach lines) overlapped with those of the CCCP control in all three conditions, consistent with MMV665807 being a protonophore. When tested at a lower concentration (0.1 μM; dashed peach lines), the trace for MMV665807 fell between those of the CCCP control and the 0.1% v/v DMSO control in the +Glucose condition. In the other two conditions, the traces of 0.1 μM MMV665807 were similar to those of CCCP. MMV687807 and MMV688991 (nitazoxanide), both from the Pathogen Box, were shown to acidify the parasite cytosol (their effects on the pH inside the DV (pH_DV_) were not tested).^13^ When tested at 1 μM, the traces for MMV687807 (dashed red lines) and MMV688991 (dashed dark red lines) fell between those of CCCP and 0.1% v/v DMSO in the +Glucose condition and overlapped with CCCP in the other two conditions. These data are consistent with MMV687807 and MMV688991 being protonophores, with their effects (when tested at 1 μM) on the H^+^ permeability of the parasite plasma membrane being lower than that of 100 nM CCCP.

**Figure 4:**
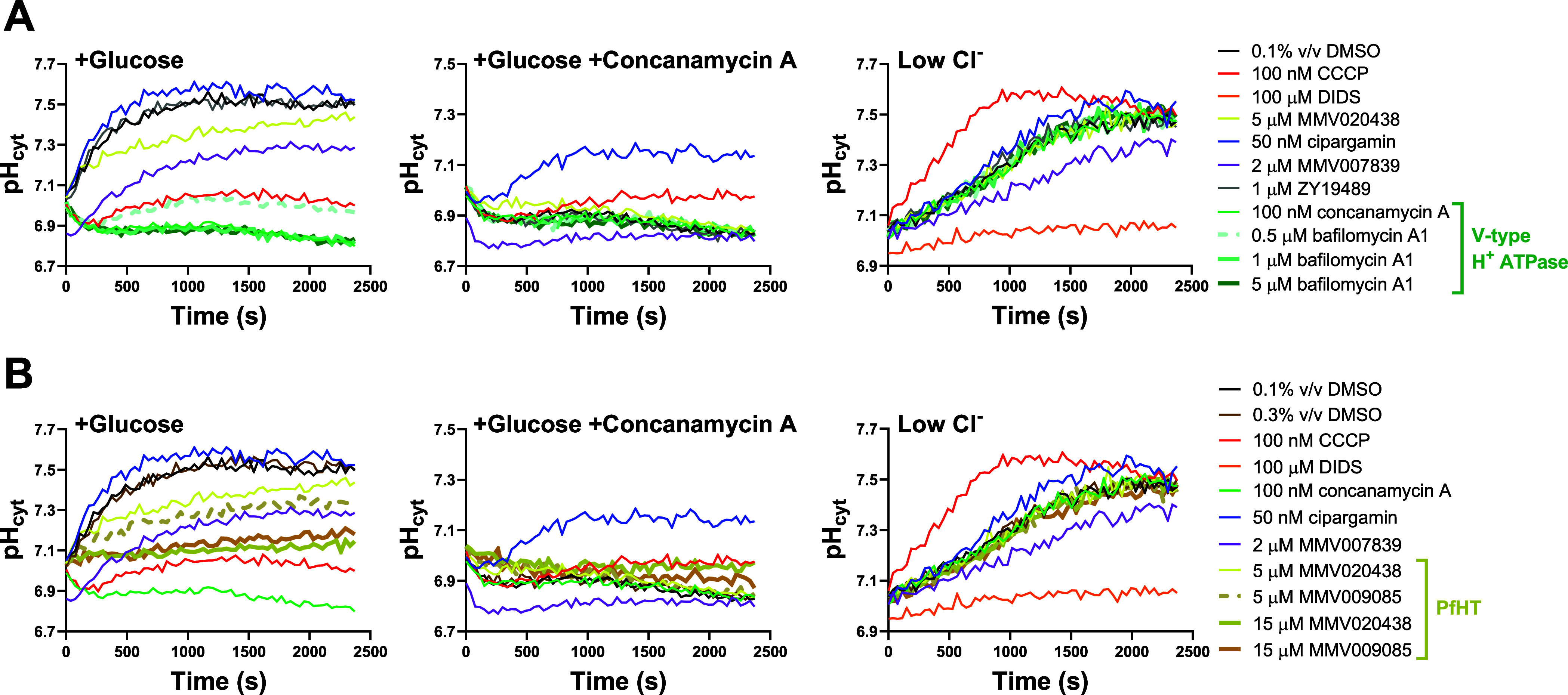
pH fingerprints of the V-type H^+^ATPase inhibitor bafilomycin A1 (at maximal and submaximal concentrations) and the clinical candidate ZY19489 (A), and the PfHT inhibitors MMV020438 and MMV009085 (B). In addition to the control compounds described in Figure 1, results for the V-type H^+^ ATPase inhibitor bafilomycin A1 (thick green/cyan traces) at 1 μM and 5 μM (which gave rise to maximal effects) and 0.5 μM (which gave rise to a submaximal effect; dashed cyan traces) and for ZY19489 (1 μM; gray traces) are shown in panel (A). Panel (B) shows results for the PfHT inhibitors MMV020438 and MMV009085 (yellow/brown lines) at concentrations of 5 and 15 μM. The effects of these compounds were greater at 15 μM (thick lines) than at 5 μM (dashed line for MMV009085, yellow line for MMV020438). When tested at 15 μM, the DMSO concentration introduced in the assay was 0.3% v/v, therefore a 0.3% v/v DMSO control (brown traces) was included in addition to the 0.1% v/v DMSO control (black traces). The data shown in the two panels are from the same experiment; thus, the traces for the control compounds are identical in each panel. All of the test compounds have been tested in at least three similar experiments, yielding consistent results (see Figure S2 for an additional experiment for each compound).

The effects of maximal (1 and 5 μM) and submaximal (0.5 μM) concentrations of the known V-type H^+^ ATPase inhibitor bafilomycin A1^34,35^ are shown in Figure 4A. Bafilomycin A1 has been shown to inhibit the growth of 3D7 parasites in 48 h assays with an IC_50_ value of 19 nM.^38^ The traces for 1 μM (thick cyan lines) and 5 μM bafiloymcin A1 (thick green lines) overlapped with those of the concanamycin A control in all three conditions. The trace for 0.5 μM bafilomycin A1 (dashed thick cyan line) fell between those of the concanamycin A and 0.1% v/v DMSO controls in the +Glucose condition (the only condition in which V-type H^+^ ATPase inhibitors can be distinguished from the 0.1% v/v DMSO control). The traces for the clinical candidate ZY19489 (1 μM; gray lines), for which resistance has been associated with mutation of the V-type H^+^ ATPase, overlapped with those of the 0.1% v/v DMSO control in all three conditions. Thus, the results of the pH fingerprint assay suggest that ZY19489 does not target the V-type H^+^ ATPase. This was investigated further (below).

The effects of the PfHT inhibitor MMV009085^49,50^ when tested at 5 μM (thick dashed brown traces) and 15 μM (thick brown traces) are shown in Figure 4B. The traces for 5 μM MMV009085 were similar to those of the 5 μM MMV020438 control, deviating from the 0.1% v/v DMSO control traces in the +Glucose and (to a lesser extent) +Glucose + concanamycin A conditions. Increasing the concentrations of MMV009085 and MMV020438 (thick yellow line) to 15 μM increased their effects in the +Glucose condition, further reducing the level of alkalinization observed. MMV009085 and MMV020438 inhibit the growth of 3D7 parasites with IC_50_ values of 0.8 and 1.2 μM, respectively, in 72 h assays.^50^

Having validated the pH fingerprint assay with additional compounds having the same modes of action as the control compounds, we set out to investigate (i) the unexpected effects of PfFNT inhibitors in the low Cl^–^ condition, and (ii) the effects of ZY19489 in additional assays in which V-type H^+^ ATPase inhibition can be detected.

### 2.3 MMV007839 Decreases the Rate of Alkalinization in the Low Cl^–^ Condition via an Effect on PfFNT

The (electroneutral) transport of anions including lactate and formate together with H^+^ by PfFNT has been investigated previously;^26,27^ however, the basis for the effects of the PfFNT inhibitors MMV007839 and MMV000972 in the low Cl^–^ condition was not clear. There was no glucose present in the low Cl^–^ condition, and it is expected that lactate production will be minimal. To investigate whether the effect of MMV007839 in the low Cl^–^ condition occurred via an interaction with PfFNT or via an “off-target effect”, we tested the two PfFNT inhibitors in pH fingerprint assays with two additional parasite lines: Dd2-PfFNT^G107S^—which acquired a G107S mutation in PfFNT in response to prolonged exposure to MMV007839 and is highly resistant to growth inhibition and lactate transport inhibition by both MMV007839 and MMV000972^6^—and its parent, Dd2-PfFNT^WT^.

**Figure 5:**
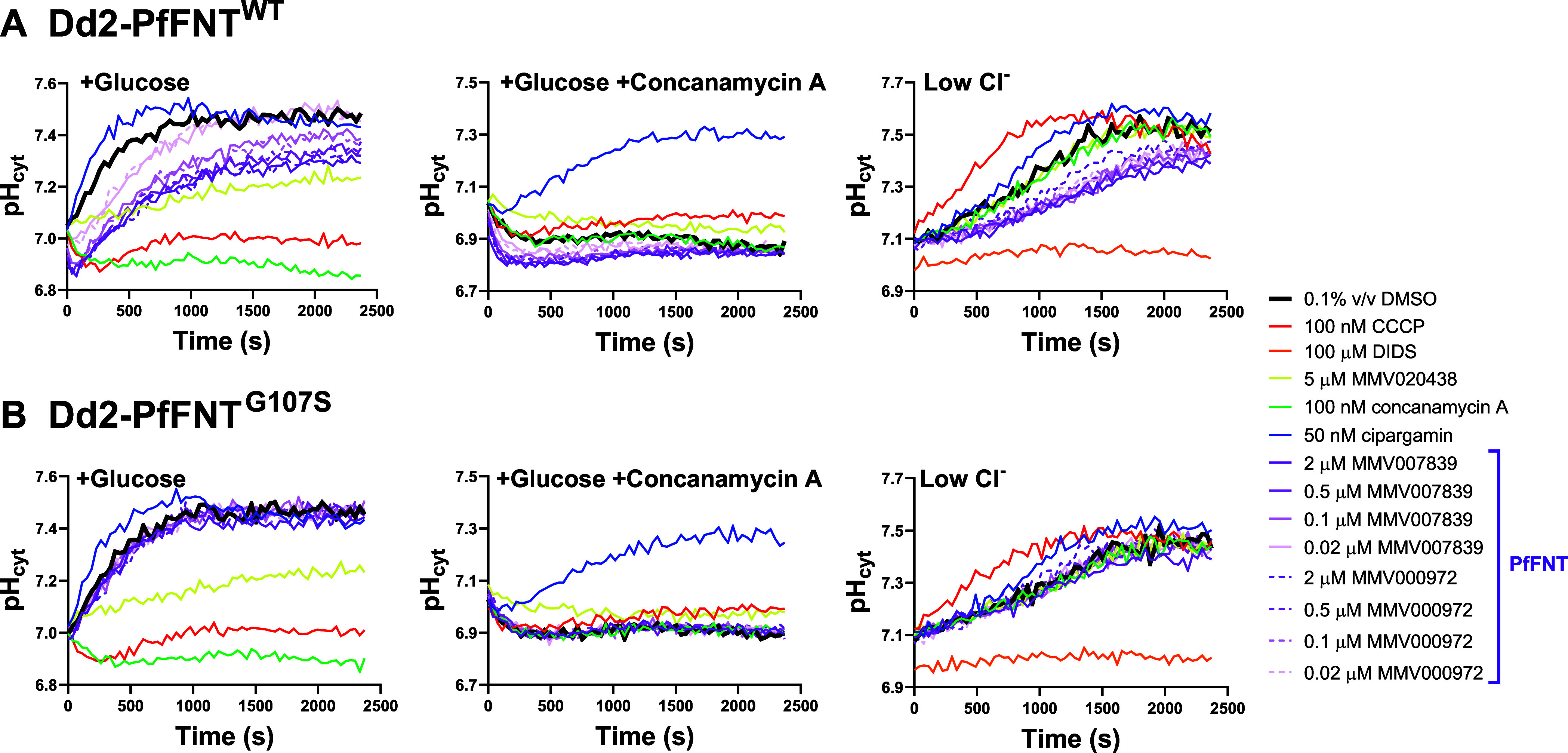
The effects of PfFNT inhibitors in pH fingerprint assays performed with MMV007839- and MMV000972-resistant Dd2-PfFNT^G107S^parasites (B) and their Dd2-PfFNT^WT^parents (A). In addition to the control compounds described in Figure 1, the PfFNT inhibitors MMV007839 (solid purple lines) and MMV000972 (dashed purple lines) were tested at concentrations of 2, 0.5, 0.1, and 0.02 μM (lighter shades for lower concentrations). The data are from a single experiment, representative of similar experiments in which the PfFNT inhibitors were tested in Dd2-PfFNT^G107S^ parasites (three experiments) and Dd2-PfFNT^WT^ parasites (two experiments).

The effects of the control compounds in the pH fingerprint assay were similar in Dd2-PfFNT^WT^ (Figure 5) and 3D7 (Figures 1–4). In Dd2-PfFNT^WT^ parasites, the PfFNT inhibitors MMV007839 and MMV000972 gave rise to the effects under all three conditions that were described above for 3D7 parasites. These effects were observed at all the concentrations tested: 2, 0.5, 0.1, and 0.02 μM, with the traces for the lowest concentration falling between those of 2 μM MMV007839 and 0.1% v/v DMSO in the +Glucose and +Glucose + concanamycin A conditions (Figure 5A), as seen for 0.02 μM MMV000972 in 3D7 in Figure 3B. Of note, MMV007839 and MMV000972 have been shown to inhibit radiolabeled lactate transport across the plasma membrane of isolated Dd2-PfFNT^WT^ parasites with IC_50_s of 158 and 49 nM, respectively.^6^

In contrast, in Dd2-PfFNT^G107S^ parasites, the traces for MMV007839 and MMV000972 were similar to those of 0.1% v/v DMSO (thick black traces) in all three conditions (Figure 5B). These findings are consistent with MMV007839 and MMV000972 exerting their effects in all three conditions via inhibition of PfFNT, and raise the possibility that PfFNT contributes to Cl^–^:H^+^ cotransport across the parasite plasma membrane. The effects of MMV007839 and MMV000972 observed with Dd2-PfFNT^WT^ parasites in the three conditions did not increase as their concentrations were increased from 0.5 to 2 μM (a concentration shown previously to fully inhibit radiolabeled lactate transport across the parasite plasma membrane^6^), consistent with 2 μM being a supramaximal concentration for both PfFNT inhibitors. This suggests that if PfFNT does contribute to Cl^–^:H^+^ transport, it is not the only protein involved, and that DIDS (a general anion transport inhibitor) likely inhibits PfFNT in addition to one or more other Cl^–^ transporter(s).

### 2.4 ZY19489 Does Not Inhibit pH Regulation by the V-Type H^+^ Pump or Its ATPase Activity

The results from our pH fingerprint assays (Figure 4A) suggested that ZY19489 does not inhibit the V-type H^+^ ATPase. To explore this finding further, we performed several different assays in which we compared the activity of ZY19489 at a range of concentrations (50 nM, 500 nM, and 5 μM) with that of the known V-type H^+^ ATPase inhibitor concanamycin A (100 nM). Two antimalarials that do not kill parasites via inhibition of the V-type H^+^ ATPase (chloroquine and dihydroartemisinin) were also included as controls.

**Figure 6:**
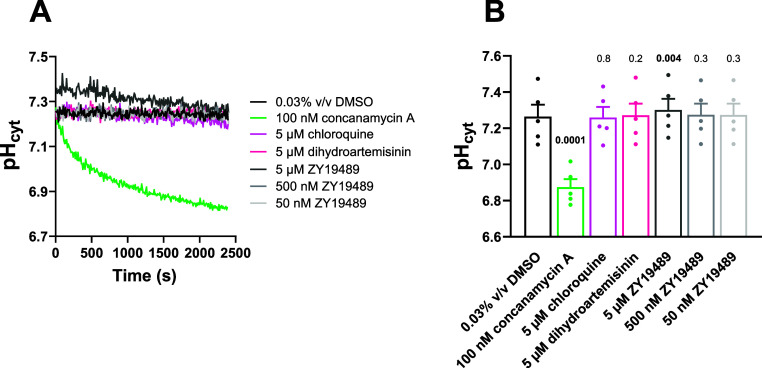
The effects of ZY19489, concanamycin A, chloroquine, and dihydroartemisinin on parasite pH_cyt_ in standard pH assays. Isolated trophozoite-stage parasites loaded with BCECF and suspended at 37 °C in physiological saline were exposed to ZY19489 (5 μM, 500 nM and 50 nM; gray). Concanamycin A (100 nM; green) served as a positive control for V-type H^+^ ATPase inhibition. DMSO (0.03% v/v; black) was the solvent control, and the antimalarials chloroquine (5 μM; light pink) and dihydroartemisinin (5 μM; dark pink) were also tested. Panel (A) shows representative traces from a single experiment. Panel (B) shows averaged data for the final pH_cyt_ reached (averaged from the data points obtained 35–40 min after parasites were first exposed to the compound/solvent) from five independent experiments performed on different days, in which all the compounds/concentrations were tested concurrently. The symbols show the data from each individual experiment. The bars and error bars show the mean + SEM. The data for each compound and concentration were compared to those for the solvent control using two-tailed paired *t* tests. The *p* values are shown above the bars, with values indicating statistical significance (*p* ≤ 0.05) shown in bold.

First, the compounds were added to isolated BCECF-loaded parasites suspended in physiological saline to examine their effects on pH_cyt_ under standard conditions. Figure 6A shows representative traces from a single experiment, and Figure 6B shows averaged data for the pH_cyt_ reached in the final 5 min of each experiment (35 min after the addition of the compound to parasites). The V-type H^+^ ATPase inhibitor concanamycin A gave rise to a pronounced acidification of the parasite cytosol under these conditions, as shown previously,^38^ whereas parasites exposed to ZY19489, chloroquine, and dihydroartemisinin did not display a decreased pH_cyt_ relative to the solvent control parasites (0.03% v/v DMSO). When tested at 5 μM, ZY19489 (a weak base) caused an initial increase in pH_cyt_, followed by a slow decline back toward normal resting pH_cyt_ (Figure 6A).

**Figure 7:**
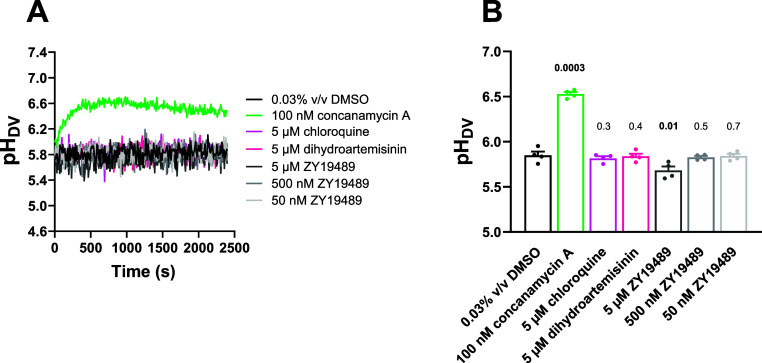
The effects of ZY19489, concanamycin A, chloroquine, and dihydroartemisinin on parasite pH_DV_. Isolated trophozoite-stage parasites containing fluorescein-dextran in their DVs and suspended at 37 °C in physiological saline were exposed to ZY19489 (5 μM, 500 nM and 50 nM; gray), concanamycin A (100 nM; green; positive control for V-type H^+^ ATPase inhibition), DMSO (0.1% v/v; black; solvent control), or the antimalarials chloroquine (5 μM; light pink) or dihydroartemisinin (5 μM; dark pink). Panel (A) shows representative traces from a single experiment. Panel (B) shows averaged data for the final pH_DV_ reached (averaged from the data points obtained 35–40 min after parasites were first exposed to the compound/solvent) from four independent experiments performed on different days in which all compounds/concentrations were tested concurrently. The bars and error bars show the mean + SEM and the symbols show the data from each individual experiment. The data for each compound and concentration were compared to those for the solvent control using two-tailed paired *t* tests. The *p* values are shown above the bars, with values indicating statistical significance (*p* ≤ 0.05) shown in bold.

We also tested the compounds for their effects on pH_DV_. These assays were performed with parasites containing fluorescein-dextran in their DVs suspended in physiological saline. Figure 7A shows representative traces from a single experiment, and Figure 7B shows averaged data for pH_DV_ reached 35–40 min after compound addition. Consistent with previous studies,^31,33^ the V-type H^+^ ATPase inhibitor concanamycin A gave rise to a pronounced alkalinization of the DV under these conditions. Parasites exposed to ZY19489, chloroquine, and dihydroartemisinin did not display an increase in pH_DV_ relative to the solvent control parasites (0.03% v/v DMSO). When tested at 5 μM, ZY19489 appeared to cause a slight but significant decrease in pH_DV_. Whether this small effect resulted from a slight optical effect of the compound at the highest concentration tested or a genuine change in pH_DV_ was not investigated.

**Figure 8:**
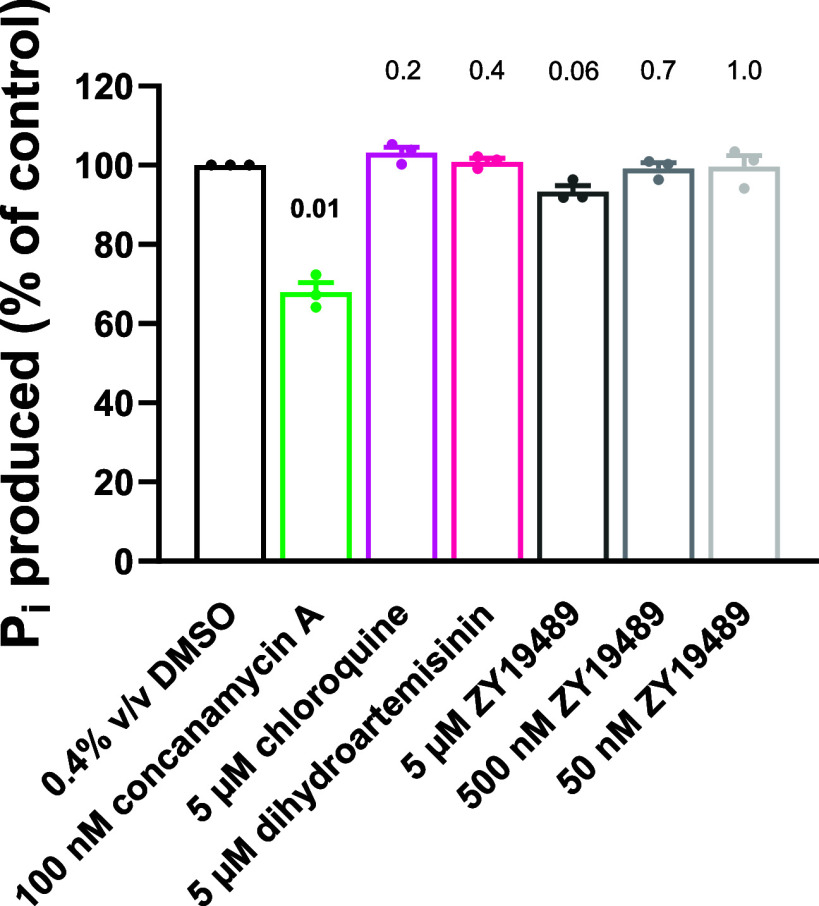
The effects of ZY19489, concanamycin A, chloroquine, and dihydroartemisinin on *P. falciparum* membrane ATPase activity under low Na^+^ conditions (2 mM Na^+^). The ATP concentration was 1 mM and the duration of the reactions was 15 min. The data were obtained with membranes prepared from isolated 3D7 trophozoites and are shown as the mean (+SEM) from three independent experiments in which all compounds/concentrations were tested concurrently, each performed on different days with different membrane preparations. The symbols show the data from individual independent experiments. Chloroquine sulfate was used in these experiments (whereas chloroquine diphosphate was used in the other experiments in this study). The (prenormalized) data for each compound and concentration were compared to those of the solvent control (0.4% v/v DMSO) using a two-tailed paired *t*-test. The *p* values are shown above the bars, with values indicating statistical significance (*p* ≤ 0.05) shown in bold.

We also examined whether any of the compounds affected ATP hydrolysis by the V-type H^+^ ATPase. We measured the production of inorganic phosphate (P_i_) from ATP in membranes prepared from isolated parasites. These reactions were performed under low Na^+^ conditions (2 mM Na^+^) to minimize the contribution of the Na^+^ pump PfATP4 to ATP hydrolysis during the experiments.^60^ Nevertheless, it is expected that multiple ATPases contribute to ATP hydrolysis in the membrane preparations. We found that concanamycin A (100 nM) decreased P_i_ production by approximately one-third (Figure 8), consistent with the V-type H^+^ ATPase making a substantial contribution to ATP hydrolysis under the conditions of the experiment. ZY19489, chloroquine, and dihydroartemisinin did not have a significant effect on ATPase activity, suggesting that none of these compounds inhibited the ATPase activity of the V-type H^+^ ATPase at the concentrations tested.

In summary, in contrast to the V-type H^+^ ATPase inhibitor concanamycin A, ZY19489 did not affect the ability of the V-type H^+^ ATPase to regulate pH_cyt_ or pH_DV_, nor did it significantly inhibit the ATPase activity attributable to the pump. To confirm that all the compounds that were tested in these assays were active, we tested their ability to inhibit parasite growth (Figure S3). ZY19489 inhibited parasite growth with an IC_50_ of 5.1 ± 0.5 nM (mean ± SEM, *n* = 3), consistent with previous findings.^40^ It was, therefore, tested at concentrations approximately 10, 100, and 1000× its IC_50_ value in the assays described above. The IC_50_s for chloroquine and dihydroartemisinin were 9.2 ± 1.4 nM and 2.1 ± 0.7 nM, respectively (mean ± SEM, *n* = 3), similar to those reported previously for the chloroquine-sensitive 3D7 strain.^61^ Concanamycin A inhibited parasite growth with an IC_50_ of 0.8 ± 0.2 nM (mean ± SEM, *n* = 3), in line with previous observations.^38^

## 3 Discussion

While target-based screens have successfully uncovered promising antimalarial lead compounds (e.g., ref (62)), many recent antimalarial candidates were identified initially based on their ability to kill asexual blood-stage *P. falciparum* parasites in culture in large-scale screens.^3,4,63^ Determining the target of an antimalarial lead compound is often challenging and time-consuming. Current approaches for target identification include in vitro evolution followed by whole genome sequencing, untargeted metabolomics, and proteomic approaches (reviewed in ref (64)). These approaches are not always successful, and there are several compounds undergoing clinical development for which the modes of action remain unknown (e.g., ref (65)). Thus, there is a need for the continual development of efficient methods for target identification.

A relatively high proportion (∼8%) of compounds identified as having the ability to kill asexual blood stage *P. falciparum* parasites show evidence of targeting PfATP4.^13,18^ The second most clinically advanced PfATP4 inhibitor after cipargamin is the structurally unrelated dihydroisoquinolone SJ733,^17^ which is undergoing testing in humans.^66,67^ With many chemically distinct PfATP4 inhibitors already identified and in different stages of development, the Malaria Drug Accelerator consortium has listed PfATP4 as a “deprioritized target”.^68^ It is important to have diversity in the antimalarial development pipeline, and pinpointing PfATP4 inhibitors at an early stage prevents over-investment in compounds sharing the same mechanism of action.

While early screens for PfATP4 inhibitors were based on the [Na^+^]_cyt_ increase that they cause, the pH fingerprint screen described here also robustly identifies PfATP4 inhibitors, is similarly inexpensive and simple to perform, and has the advantage of allowing compounds to be tested for their effects on other potential targets simultaneously. PfATP4 inhibitors are readily detectable through the alkalinization that they give rise to in the +Glucose + concanamycin A condition. Normally, parasites undergo a slight acidification in this condition, likely as a result of PfATP4-mediated acid loading.^52^ Inhibition of PfATP4 prevented this acid loading from occurring. Thus, it is to be expected that the pH_cyt_ does not decrease in the presence of a PfATP4 inhibitor in the +Glucose + concanamycin A condition. To fully understand why pH_cyt_ increases, we would need a more complete understanding of H^+^ consuming/efflux and producing/importing pathways and their regulation. A variety of secondary assays can be used to confirm PfATP4 inhibition after hit identification in the pH fingerprint assay: PfATP4 inhibitors give rise to an increase in [Na^+^]_cyt_,^9^ a Na^+^-dependent increase in parasite and host erythrocyte volume,^11^ and inhibition of Na^+^-ATPase activity in parasite membrane preparations.^9,13,60^

PfFNT inhibition also gives rise to a unique, readily identifiable pH fingerprint. The effects of PfFNT inhibitors in the +Glucose and + Glucose + concanamycin A conditions can be understood based on the detailed information that exists on PfFNT’s function.^6,26,27^ A variety of secondary assays are available that could be used to confirm PfFNT inhibition by a test compound, including measurements of radiolabeled lactate transport into parasites,^6^ or into *Xenopus* oocytes or yeast expressing PfFNT,^6,22^ and measurements of parasite pH_cyt_ when lactate is added to the medium (creating an inward lactate gradient and a consequent acidification).^6^ Thus, far, MMV007839 and MMV000972, which are structurally very similar to one another, and other structural derivatives synthesized by Beitz and colleagues^22,23^ are the only PfFNT inhibitors identified to date. MMV007839 and MMV000972 displayed toxicity against mammalian cells in some assays,^59^ and rapidly selected for highly resistant parasites with a G107S mutation in PfFNT.^6,22^ Thus, discovering additional PfFNT-targeting chemotypes with greater selectivity for parasites that are less prone to resistance would be desirable. Recent high-resolution structures of PfFNT^24,25^ may prove useful in this regard.

The unexpected effects of PfFNT inhibitors in the low Cl^–^ condition highlighted the fact that there is more to learn about the transport properties and regulation of the transporters shown in Figure 1A. The data presented here raise the possibility that PfFNT makes a contribution to Cl^–^:H^+^ transport across the plasma membrane. Of note, a member of the FNT family in *Salmonella typhimurium* (FocA) has been shown to mediate the passage of Cl^–^ across membranes.^69^ Further studies on PfFNT-mediated Cl^–^ transport are eagerly awaited.

Even if PfFNT does serve as a Cl^–^:H^+^ transporter, there is likely to be an additional acid-loading Cl^–^ transporter to discover, as concentrations of MMV007839 and MMV000972 that fully inhibit lactate transport by PfFNT did not completely inhibit the alkalinization observed upon the creation of an outward Cl^–^ gradient across the parasite plasma membrane. With the exception of the broad-specificity anion transport inhibitor DIDS, we are yet to find a compound that inhibits the alkalinization normally observed in Low Cl^–^ to a greater extent than that caused by PfFNT inhibitors. Cl^–^ transport into the parasite is likely to be an essential process; thus, finding such a compound may provide a means to determine the identity of the (other) Cl^–^ transporter through in vitro evolution studies. Secondary assays that could be used to confirm that a test compound inhibits Cl^–^ transport could include measurements of (radioactive) ^36^Cl^–^ transport into parasites and monitoring of parasite cytosolic [Cl^–^] using the dye MQAE (as described in ref (48)).

The pH fingerprint of V-type H^+^ ATPase inhibitors was expected based on what is known about the function of this pump—it serves as the parasite’s primary regulator of pH_cyt_, allowing parasites to establish an inward H^+^ gradient across their plasma membrane.^28^ The highly conserved V-type H^+^ ATPase plays a variety of important roles in human cells too, acidifying lysosomes and neurotransmitter vesicles, and effluxing H^+^ into the external environment across the plasma membrane in specialized cell types such as osteoclasts.^70^ Nevertheless, the V-type H^+^ ATPase is being investigated as a drug target for several diseases. Some types of cancer cells are over-reliant on V-type H^+^ ATPase activity, and compounds targeting the pump have shown promise in inhibiting the growth of certain tumors in mice.^71,72^ The V-type H^+^ ATPase is also being investigated as a therapeutic target for osteoporosis.^73^ A variety of secondary assays are available to further investigate antiplasmodial compounds should they be pinpointed as V-type H^+^ ATPase inhibitors in the pH fingerprint assay, including those that were used to investigate ZY19489 in this study: measurements of pH_DV_ and membrane ATPase activity.

Our experiments with ZY19489 suggested that the compound does not kill parasites via inhibition of H^+^ pumping or ATP hydrolysis by the V-type H^+^ ATPase. However, it remains possible that the compound inhibits an aspect of the V-type H^+^ ATPase’s function that was not investigated here (e.g., an interaction with protein(s) involved in cell signaling). Furthermore, even if ZY19489 does not affect any aspect of the function of the V-type H^+^ ATPase, it remains possible that the mutation in the D subunit of the V-type H^+^ ATPase (G29V; observed in parasites with low-level resistance to ZY19489^40^) plays a role in reduced parasite susceptibility to ZY19489. For example, if the mutation modulates the activity of the V-type H^+^ ATPase, this might affect the physiological environment within the parasite cytosol or DV in such a way as to alter the accumulation of ZY19489. Hameed P et al.^40^ reported that the DVs of parasites with the G29V mutation were larger than those of their wild-type parents, the significance of which is not yet clear. Similarly, the mechanism by which mutations in the A subunit of the V-type H^+^ ATPase affect parasite susceptibility to the antiplasmodial compound SQ109^41^ has not been established and remains to be determined.

PfHT inhibitors indirectly inhibit the V-type H^+^ ATPase, reducing the parasite’s ability to recover an alkaline resting pH_cyt_ in the +Glucose condition. However, the traces for PfHT inhibitors do not overlay those of V-type H^+^ ATPase inhibitors (at least when the latter are used at maximal concentrations). This is likely because PfATP4-mediated acid-loading gives rise to an acidification under the +Glucose condition when the V-type H^+^ ATPase is fully inhibited. By depriving parasites of glucose, PfHT inhibitors are expected to inhibit PfATP4 as well as the V-type H^+^ ATPase. Nevertheless, inhibitors of glycolysis would be expected to give rise to pH fingerprints resembling those of PfHT inhibitors, and secondary assays would be essential to determine whether a test compound is a PfHT inhibitor. These could include measurements of the transport of radiolabeled glucose or nonmetabolisable glucose analogues into isolated parasites or other cell types engineered to express PfHT (as in e.g., refs (43, 49, 50 and 74)).

Three compounds from the Malaria Box and Pathogen Box that were found to dissipate the H^+^ gradient across the parasite plasma membrane (acidifying the cytosol^6,13^) were found to behave like the well-characterized protonophore CCCP in the pH fingerprint assay, suggesting that the pH fingerprint assay can diagnose compounds that have protonophore activity. One of these (MMV665807) was also shown to dissipate the H^+^ gradient across the DV membrane,^6^ while another, the antiparasitic drug nitazoxanide (MMV688991, a reference compound in the Pathogen Box), has been found to disrupt pH and membrane potential in *Mycobacterium tuberculosis* in a similar way to CCCP.^75^ Understanding the effect of protonophores in the low Cl^–^ condition would require knowledge of the membrane potential under the conditions of the experiment. It is possible that under the conditions of the experiment (in which there is no V-type H^+^ ATPase activity to create an inwardly negative membrane potential), the membrane potential is influenced by the Cl^–^ equilibrium potential which, under the low extracellular Cl^–^ concentration conditions prevailing here will be inwardly positive, creating an outward H^+^ electrochemical gradient. If this were the case, increasing the H^+^ permeability of the plasma membrane with CCCP would be expected to increase the rate at which H^+^ ions exit the cell (i.e., increase the rate of alkalinization, as was observed). It would be interesting in the future to examine the effects of other compounds that might influence the membrane potential (e.g., K^+^ ionophores) in this condition. Test compounds tentatively identified as protonophores in the pH fingerprint assay could be investigated in pH_DV_ assays to determine whether they also dissipate the H^+^ gradient across the DV membrane.

Our study has limitations. The pH fingerprint assay is performed with whole cells, and it is possible that a compound that appears to inhibit one of the H^+^ transporters actually inhibits a protein involved in regulating the activity of that transporter. Furthermore, even if a compound does inhibit a particular transporter (or possesses protonophore activity), further investigation is required to determine whether this constitutes the primary mechanism by which the compound kills parasites. In the case of compounds found to inhibit a particular transporter, testing their parasite killing potencies in parasite lines in which the transporter is overexpressed or knocked down can provide further information on whether the transporter is indeed the primary target of the compound.^68^

It may be possible to improve the assay further in future. The assay could be adapted to a 384-well format. This would likely come at a cost to the time resolution, but specific time points could be chosen in which all of the different control compounds can be distinguished. It is also possible that additional H^+^ transporters on the plasma membrane, or other proteins that impact pH_cyt_ under certain conditions, will be discovered and that these could be built into an expanded assay in the future. When used on a large scale, it is possible that compounds will be discovered that give rise to new pH fingerprints, which may, in turn, lead to the discovery of novel targets involved in pH regulation in asexual blood-stage *P. falciparum* parasites.

## 4 Conclusions

We have developed a pH-based assay that has the potential to detect and discriminate between inhibitors of PfATP4, PfFNT, the V-type H^+^ ATPase, the acid-loading Cl^–^ transporter(s), PfHT/glycolysis, and compounds with protonophore activity. Our data generated with this assay raised the possibility that PfFNT contributes to Cl^–^ transport in the parasite and revealed that the clinical candidate ZY19489 does not act by inhibiting H^+^ transport or ATP hydrolysis by the V-type H^+^ ATPase. The pH fingerprint assay could serve as a useful additional tool in the antimalarial drug development pipeline.

## 5 Materials and Methods

### 5.1 Use of Human Blood

The use of human blood (from anonymous O+ donors) in this study was approved by the Australian National University Human Research Ethics Committee (Protocol numbers 2011/266 and 2017/351).

### 5.2 Compounds

Some of the compounds used in this study were from MMV’s Pathogen Box (MMV687807, MMV688991, and the compounds for which data are shown in Figure S1). Cipargamin, SJ733 (the (+) enantiomer), MMV020438, MMV009085, MMV665807, and ZY19489 were kindly provided as powders by MMV. PA21A050 was kindly provided by Assoc. Prof. Erkang Fan and Prof. Akhil Vaidya. MMV007839 and MMV000972 were purchased from MolPort, dihydroartemisinin was purchased from Selleck Chemicals, and concanamycin A and bafilomycin A1 were obtained from Sapphire Biosciences. All other compounds were from Sigma-Aldrich.

### 5.3 pH Fingerprint Assay

The pH fingerprint assay was carried out in a 96-well plate format, using clear flat-bottom 96-well plates. Test compounds and control compounds (dissolved in DMSO at 1000× the desired final concentrations) were first diluted 1 in 25 in “Glucose-free Saline” (135 mM NaCl, 5 mM KCl, 1 mM MgCl_2_, 25 mM HEPES; pH 7.10). Aliquots (5 μL) of each diluted compound were then added to 3 separate wells in the same column but adjacent rows of a 96 well plate. Three different solutions were then added to different rows, with 180 μL added to each individual well, in order that each compound be tested under three different conditions. The three solutions were: (1) “Physiological Saline” (125 mM NaCl, 5 mM KCl, 1 mM MgCl_2_, 20 mM glucose, 25 mM HEPES; pH 7.1; to create the +Glucose condition), (2) Physiological Saline to which concanamycin A had been added (from a 1000× DMSO stock, yielding a final concentration in the assay of 100 nM; to create the +Glucose + concanamycin A condition), and (3) “Cl^–^-free Glucose-free Saline” (135 mM Na^+^-gluconate, 5 mM K^+^-gluconate, 1 mM MgSO_4_, 25 mM HEPES; pH 7.10; to create the low Cl^–^ condition). A sticker was placed on the wells to prevent evaporation, and the plate contents were warmed to 37 °C while the cells were prepared.

Trophozoite-stage parasites (the 3D7 strain, except for the experiments shown in Figure 5 for which the Dd2-PfFNT^WT^ and Dd2-PfFNT^G107S^ lines were used; all cultured and synchronized as described previously^6^) were isolated from their host erythrocytes via brief exposure of a 50 mL culture (∼4% hematocrit, 5–10% parasitaemia) to saponin (final concentration 0.05% w/v, of which ≥10% was the active agent sapogenin). The parasites were centrifuged at 1000*g* for 5 min, then the supernatant medium was discarded and the parasites were resuspended and washed several times (with 12,000*g*, ∼30 s centrifugation steps) in “bicarbonate-free medium” (bicarbonate-free RPMI 1640 (Sigma R6504) supplemented with 11 mM additional glucose, 0.2 mM hypoxanthine and 25 mM HEPES; pH 7.1). The parasites were then resuspended in 1–2 mL of bicarbonate-free medium, and the pH-sensitive fluorescent dye BCECF-AM (Thermo Fisher Scientific B1170; dissolved in DMSO at 1 mM) was added to give a final concentration of 5 μM. The parasites were incubated for 10 min at 37 °C to allow for dye loading. The parasites were then washed several times (with 12,000*g*, ∼30 s centrifugation steps) in Glucose-free Saline, then resuspended in this solution and incubated for 20 min at 37 °C to allow sufficient time for ATP depletion to occur. The procedures for dye loading and ATP depletion are similar to those described previously.^28^

During the 20 min incubation step, four 15 μL aliquots of the parasite suspension were centrifuged (12,000*g* for ∼30 s), and the supernatant solution removed. The parasites were then suspended in 200 μL of a “pH Calibration Saline” (130 mM KCl, 1 mM MgCl_2_, 20 mM glucose, 25 mM HEPES; pH 6.8, 7.1, 7.4, or 7.8) to which nigericin had been added (final concentration of 5 μM, added from a 5 mM stock in DMSO/ethanol 1:1). Nigericin facilitates H^+^/K^+^ exchange, thereby causing pH_cyt_ to become the same as the pH of the external solution.^28^ The 200 μL parasite suspensions were then transferred to wells of a 96-well plate, and fluorescence was monitored for 3–5 min at 37 °C on a TECAN Infinite M1000 PRO plate reader with i-control (version 1.12) software. The fluorophore was excited successively at wavelengths of 440 (yielding pH-insensitive fluorescence) and 495 nm (yielding pH-sensitive fluorescence), with the emission recorded at 520 nm.

After the 20 min incubation step, 15 μL aliquots of the parasites suspended in Glucose-free Saline were added to wells of the test plate containing the different solutions and compounds of interest, yielding a final volume of 200 μL in each well, and fluorescence measurements were commenced as quickly as possible, at 37 °C and typically for 40 min, using the plate reader and wavelengths described above.

Changes in the fluorescence ratio (495/440 nm) are indicative of changes in pH_cyt_. To convert fluorescence ratio values to pH_cyt_ values, we averaged the fluorescence ratio values obtained over the 3–5 min read for the parasites suspended in pH Calibration Salines, and we plotted the average value against the pH of the solution, which showed a linear relationship. A line was fit to the data (fluorescence ratio = *m* × pH_cyt_ + *c*), where *m* and *c* are the gradient and *y*-intercept, respectively. The equation was rearranged, and the values for *m* and *c* used to convert fluorescence ratio values to pH_cyt_.

### 5.4 Secondary Assays Performed with ZY19489

#### 5.4.1 pH_cyt_ Experiments (Standard Conditions with No ATP Depletion)

Trophozoite-stage parasites (3D7 strain) were isolated and loaded with BCECF as described above, then washed twice in bicarbonate-free medium and once in Physiological Saline to remove extracellular dye. The parasites were then suspended in either Physiological Saline containing the compound of interest (or solvent alone; 0.03% v/v DMSO) or a pH Calibration Saline (pH 6.8, 7.1, 7.4 or 7.8, containing 30 μM nigericin), and fluorescence was measured at 37 °C then converted to pH_cyt_ as described above.

#### 5.4.2 Measurements of pH_DV_

Parasites (3D7 strain) containing fluorescein-dextran (10,000 MW; Molecular Probes) in their DVs were prepared as described previously,^76^ with ∼10 μM fluorescein present in the loading solution. Trophozoite-stage parasites were isolated from their host erythrocytes as described above then washed twice in Physiological Saline, before being washed and resuspended in either Physiological Saline or a high-K^+^ calibration saline containing nigericin (30 μM) (the nigericin/high-K^+^ calibration approach described by Hayward et al.^31^ was used to convert fluorescence ratio into pH_DV_). Fluorescence was measured at 37 °C using the same instrument and wavelengths described above.

#### 5.4.3 Membrane ATPase Assays

Compounds of interest were tested for their effects on membrane ATPase activity under low Na^+^ conditions (to minimize the contribution of PfATP4 to ATPase activity). Membranes were prepared from saponin-isolated trophozoite-stage parasites (3D7 strain), their protein content determined using a Bradford assay, and ATPase activity measured using a PiColorLock Gold Phosphate Detection System (Innova Biosciences), as described previously.^60^ The final reactions contained 150 mM choline chloride, 50 mM Tris, 20 mM KCl, 2 mM MgCl_2_, 50 μg/mL (total) membrane protein, the compound of interest or solvent alone (introducing 0.4% v/v DMSO), and 1 mM ATP (Na_2_ATP.3H_2_O; MP Biomedicals) and had a pH of 7.2. The reactions were performed over 15 min at 37 °C.

#### 5.4.4 Parasite Proliferation Assays

Parasite proliferation assays were initiated with cultures containing predominantly ring-stage parasites with a parasitemia of 1% and a hematocrit of 1%. The duration of the assays was 72 h and parasite growth was assessed using the DNA-intercalating dye SYBR Safe, essentially as described previously.^77,78^

## Supporting Information Available

Screen of Pathogen Box for PfATP4 inhibitors; additional biological replicates for compounds tested in the pH fingerprint assay; and determination of the antiplasmodial activity of ZY19489 and control compounds (PDF)

## Abbreviations

BCECF-AM: 2′,7′-bis(2-carboxyethyl)-5-(and-6)-carboxyfluorescein, acetoxymethyl ester
CCCP: carbonyl cyanide *m*-chlorophenyl hydrazone
DIDS: 4,4′-diisothiocyano-2,2′-stilbenedisulfonic acid
DV: digestive vacuole
HEPES: 2-[4-(2-hydroxyethyl)piperazin-1-yl]ethanesulfonic acid
MMV: Medicines for Malaria Venture
[Na^+^]_cyt_: cytosolic Na^+^ concentration
PfFNT: *Plasmodium falciparum* formate nitrite transporter
PfHT: *Plasmodium falciparum* hexose transporter
pH_cyt_: cytosolic pH
pH_DV_: digestive vacuole pH
RPMI: Roswell Park Memorial Institute
