# Supplementary material for: A pH Fingerprint Assay to Identify Inhibitors of Multiple Validated and Potential Antimalarial Drug Targets
Source: ACS Infect Dis. 2024 Mar 18;10(4):1185–200. doi: 10.1021/acsinfecdis.3c00588 (PMC11019546; doi:10.1021/acsinfecdis.3c00588)
Supplement: Supplementary file 1 — id3c00588_si_001.pdf [file id3c00588_si_001.pdf]

A pH fingerprint assay to identify inhibitors of multiple validated and potential antimalarial drug targets

Julia C. R. Lindblom[¶], Xinxin Zhang[¶], Adele M. Lehane*

Research School of Biology, Australian National University, Canberra, ACT 2600, Australia

*Corresponding author; e-mail: adele.lehane@anu.edu.au

[¶]These authors contributed equally to this work.

Supporting Information

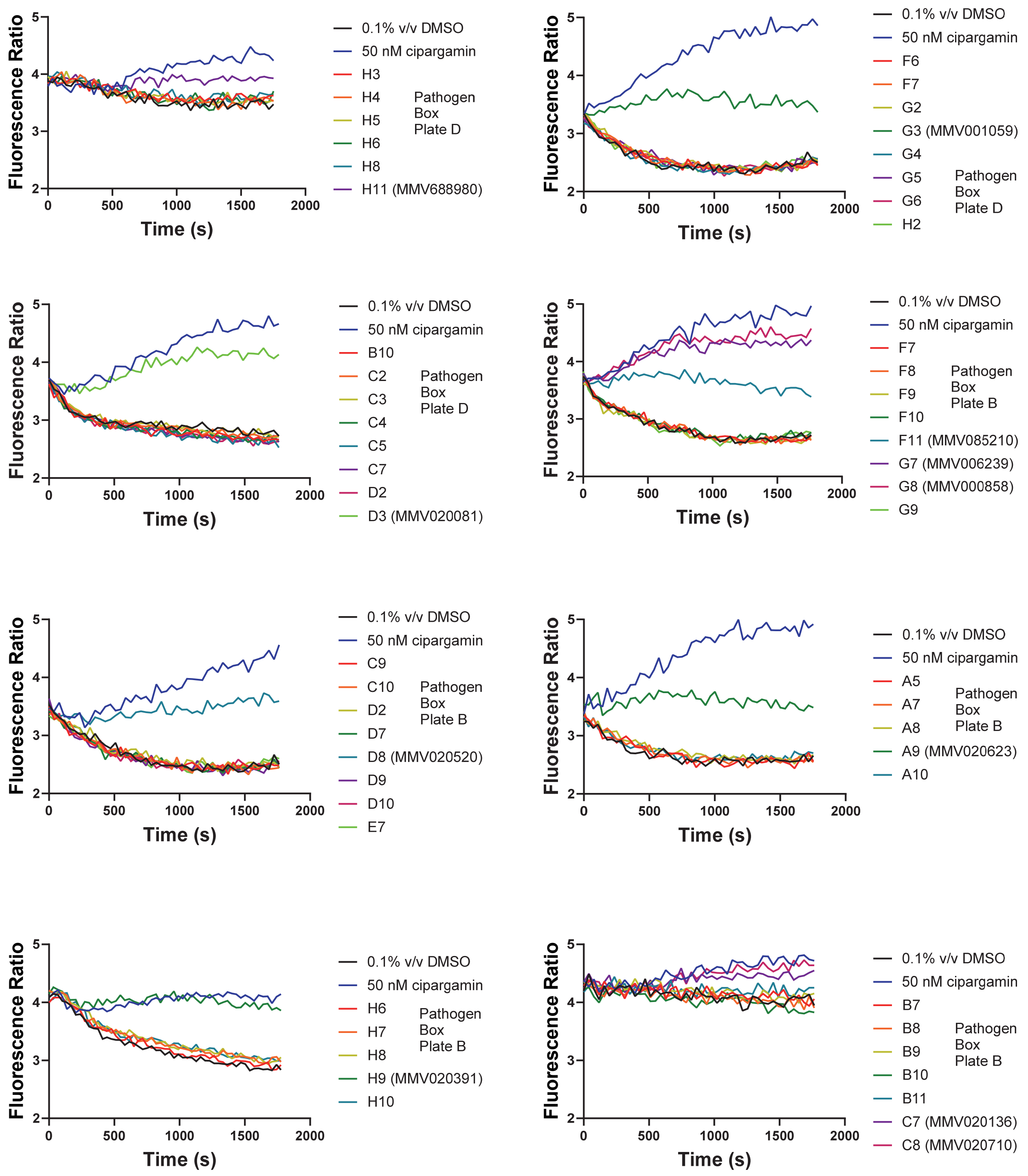

Figure S1. Identification of PfATP4 inhibitors in a blind screen of the antiplasmodial Pathogen Box compounds based on their effects in the +Glucose +Concanamycin A condition of the pH fingerprint screen. *The Pathogen Box compounds were tested at a concentration of 1 µM. For compounds that did not show evidence for PfATP4 inhibition, their well numbers in the relevant Pathogen Box plate are indicated in the legends. For PfATP4 'hits', their well numbers and compound names are indicated. These experiments were not calibrated; an increase in Fluorescence Ratio is indicative of an increase in* pH_{cyt}*. DMSO (0.1% v/v; black traces) served as the solvent control and cipargamin (50 nM; blue traces) was included as a positive control for PfATP4 inhibition.*

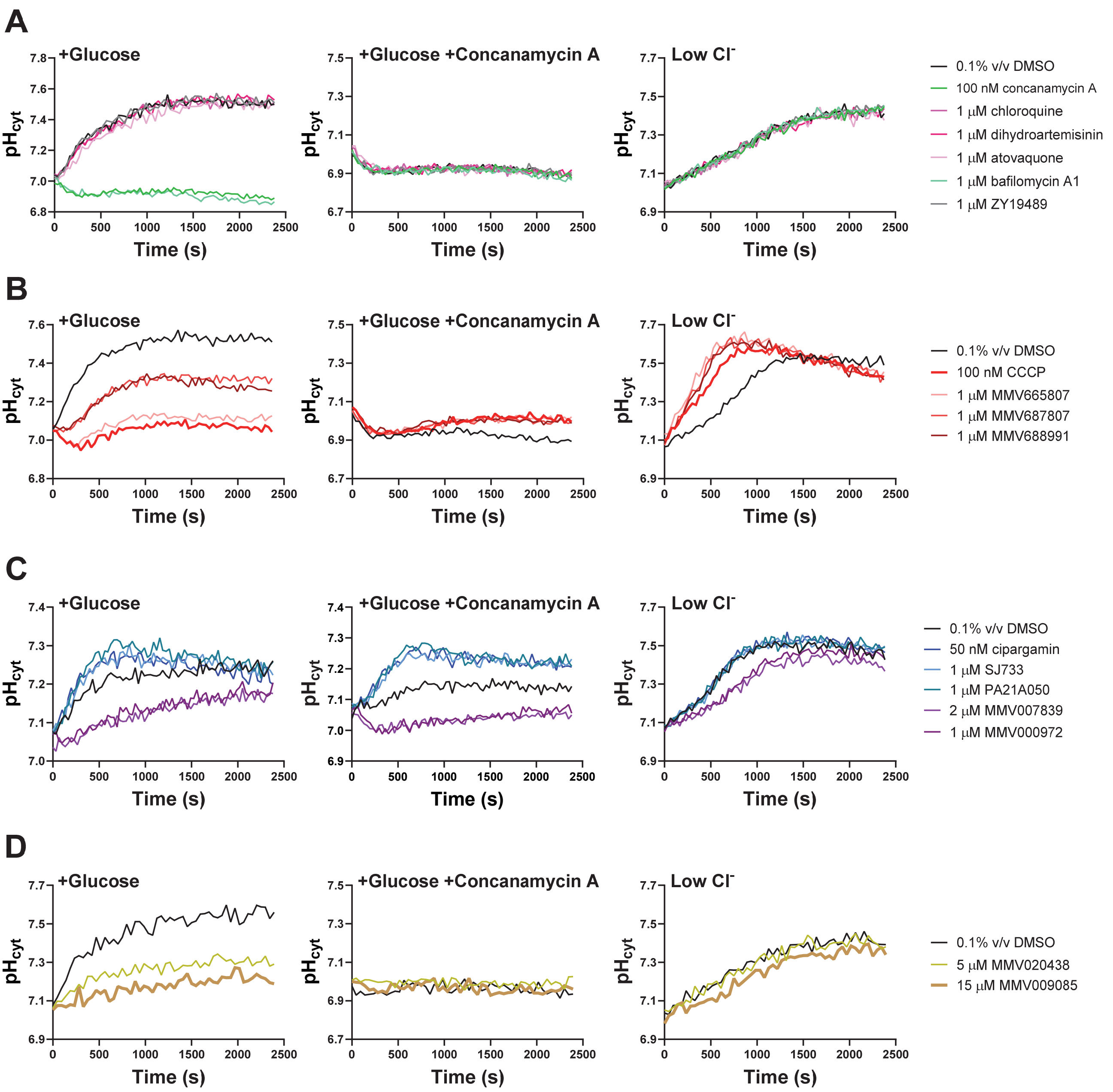

Figure S2. Additional biological replicates showing the effects of compounds with different modes of action in the pH fingerprint assay. *(**A**) Traces for the antimalarials chloroquine (1 µM; pink), dihydroartemisinin (1 µM; dark pink), atovaquone (1 µM; light pink) and ZY19489 (1 µM; grey), which do not have any of the mechanisms of action that can be detected with the assay, and the V-type H^+ ATPase inhibitor bafilomycin A1 (1 µM; light green). The solvent (0.1% v/v DMSO; black traces) and V-type H^+ ATPase inhibitor (100 nM concanamycin A; green traces) controls are shown for comparison. (**B**) Traces for the suspected protonophores MMV665807 (1 µM; peach), MMV687807 (1 µM; red), and MMV688991 (1 µM; dark red). The solvent (0.1% v/v DMSO; black traces) and protonophore (100 nM CCCP; thick red traces) controls are shown for comparison (note that the traces for these two control*

compounds are identical to those shown in ***Fig. 2****, as the protonophores were tested in the same experiment for which data for the PfATP4 inhibitors are shown in* ***Fig. 2****). (****C****) Traces for the PfATP4 inhibitors SJ733 (1 μM; light blue) and PA21A050 (1 μM; turquoise) and the PfFNT inhibitor MMV000972 (1 μM; dark purple). The solvent (0.1% v/v DMSO; black traces), PfATP4 inhibitor (50 nM cipargamin; blue traces) and PfFNT inhibitor (2 μM MMV007839; purple traces) controls are shown for comparison. (****D****) Traces for the PfHT inhibitor MMV009085 (tested here at 15 μM in 0.3% v/v DMSO; thick brown lines), the PfHT inhibitor control MMV020438 (5 μM; yellow lines) and the solvent control (0.1% v/v DMSO; black lines). Note that the y-axis ranges vary in the different graphs in panels* ***A****-****D****.*

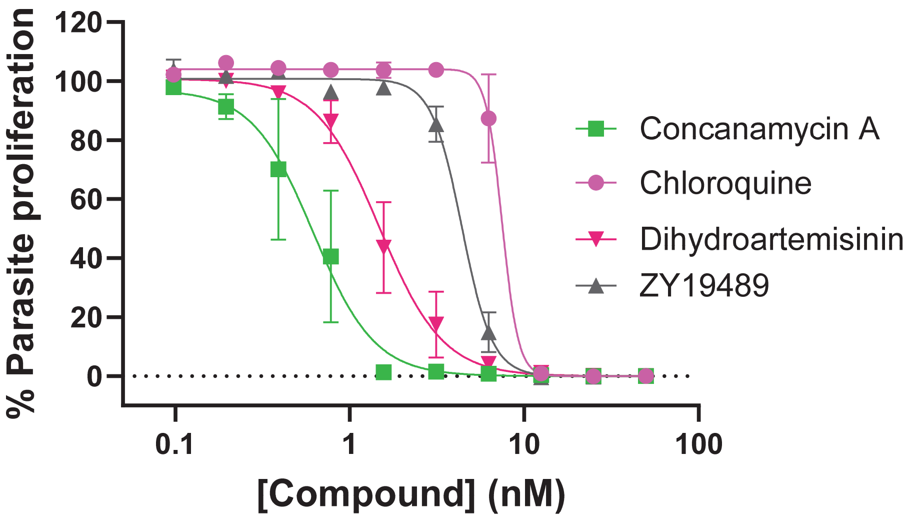

Figure S3. Inhibition of **P. falciparum** ***proliferation by ZY19489 (grey), concanamycin A (green), chloroquine (light pink) and dihydroartemisinin (dark pink).*** *Cultures containing 3D7 parasites were exposed to a range of concentrations of each of the compounds indicated for 72 h. The DMSO concentration that the parasites were exposed to did not exceed 0.04%. The data show the average (± SEM) from three independent experiments performed on different days.*
